# Predictors of Long COVID in Patients without Comorbidities: Data from the Polish Long-COVID Cardiovascular (PoLoCOV-CVD) Study

**DOI:** 10.3390/jcm11174980

**Published:** 2022-08-25

**Authors:** Michał Chudzik, Joanna Lewek, Joanna Kapusta, Maciej Banach, Piotr Jankowski, Agata Bielecka-Dabrowa

**Affiliations:** 1 Department of Internal Medicine and Geriatric Cardiology, Medical Centre of Postgraduate Education, 01-813 Warsaw, Poland; 2 Department of Cardiology and Congenital Diseases of Adults, Polish Mother’s Memorial Hospital Research Institute (PMMHRI), 93-338 Lodz, Poland; 3 Department of Preventive Cardiology and Lipidology, Medical University of Lodz (MUL), 93-338 Lodz, Poland; 4 Department of Internal Medicine and Cardiac Rehabilitation, Medical University of Lodz, 70-445 Lodz, Poland; 5 Department of Epidemiology and Health Promotion, School of Public Health, Medical Centre of Postgraduate Education, 01-826 Warsaw, Poland

**Keywords:** COVID-19, COVID complications, Long COVID, chronic fatigue syndrome

## Abstract

Background: The SARS-CoV-2 pandemic has become an enormous worldwide challenge over the last two years. However, little is still known about the risk of Long COVID (LC) in patients without comorbidities. Thus, we aimed to assess the predictors of LC in patients without comorbidities. Methods: Patients’ information, the course of the disease with symptoms, and post-COVID-19 complaints were collected within 4–12 weeks after COVID-19 recovery. Next, the patients were followed for at least 3 months. ECG, 24-h ECG monitoring, 24-h blood pressure (BP) monitoring, echocardiography, and selected biochemical tests were performed. LC was recognized based on the WHO definition. Results: We identified 701 consecutive patients, 488 of whom completed a 3-month follow-up (63% women). Comparisons were made between the LC group (*n* = 218) and patients without any symptoms after SARS-CoV-2 recovery (non-LC group) (*n* = 270). Patients with a severe course of acute-phase COVID-19 developed LC complications more often (34% vs. 19%, *p* < 0.0001). The persistent symptoms were observed in 45% of LC patients. The LC group also had significantly more symptoms during the acute phase of COVID-19, and they suffered significantly more often from dyspnoea (48 vs. 33%), fatigue (72 vs. 63%), chest pain (50 vs. 36%), leg muscle pain (41 vs. 32%), headache (66 vs. 52%), arthralgia (44 vs. 25%), and chills (34 vs. 25%). In LC patients, significant differences regarding sex and body mass index were observed—woman: 69% vs. 56% (*p* = 0.003), and BMI: 28 [24–31] vs. 26 kg/m2 [23–30] (*p* < 0.001), respectively. The number of symptoms in the acute phase was significantly greater in the LC group than in the control group (5 [2–8] vs. 2 [1–5], *p* = 0.0001). The LC group also had a higher 24-h heart rate (77 [72–83] vs. 75 [70–81], *p* = 0.021) at admission to the outpatient clinic. Multivariate regression analysis showed that LC patients had a higher BMI (odds ratio (OR): 1.06, 95% confidence intervals [CI]: 1.02–1.10, *p* = 0.007), almost twice as often had a severe course of COVID-19 (OR: 1.74, CI: 1.07–2.81, *p* = 0.025), and presented with joint pain in the acute phase (OR: 1.90, CI: 1.23–2.95, *p* = 0.004). Conclusions: A severe course of COVID-19, BMI, and arthralgia are independently associated with the risk of Long COVID in healthy individuals.

## 1. Introduction

According to the World Health Organization (WHO), the coronavirus disease 2019 (COVID-19) disease has infected nearly 250 million people and has caused 6 million deaths; however, it seems that this number is even twice underestimated [1]. COVID-19 is a serious respiratory disease that may affect many other organs, and its consequences may also appear weeks after recovery in the form of Long COVID syndrome. There is still not enough data regarding early and late complications of SARS-CoV-2 infection. Patients who have recovered from SARS-CoV-2 infection complain of persisting symptoms including diffuse myalgia, fatigue, and weakness, as well as many other complications [1,2,3,4,5].

In the Italian observation of 143 patients, after hospitalization due to COVID-19, more than 87% of cases showed persistent symptoms, i.e., fatigue (53%), dyspnea (43%), joint pain (27%), and chest pain (22%) lasting at least 60 days from the initial onset of infection. In a 6-month cohort study with 1733 Chinese patients, the authors reported persistent fatigue and muscle weakness (63%), as well as sleep disturbances (26%) and anxiety and depression (23%) in patients after COVID-19 recovery [3]. Symptoms can arise in any system, including cardiovascular, respiratory, digestive, nervous, and musculoskeletal, and include psychiatric problems, generalized pain, fatigue, and persistent fever [6]. Research shows that approximately 10–30% of patients who have recovered from COVID-19 experience persistent symptoms even several months after the acute disease [3]. Post-COVID-19 syndrome occurs in all patients, both with mild and severe COVID-19, and it depends on the severity of symptoms in the acute phase of the disease [5].

According to the WHO definition, Long COVID occurs in patients with a history of SARS-CoV-2 infection, usually 3 months (12 weeks) after COVID-19 presentation, with symptoms that last for at least 8 weeks and cannot be explained by an alternative diagnosis [7]. Symptoms may be a new onset following initial recovery from an acute COVID-19 episode or persist from the initial illness. Symptoms may also fluctuate or relapse over time [7]. The Center for Disease Control and Prevention (CDC) gives a similar definition, with different time frames, exceeding 4 weeks after acute illness [8]. The British National Institute for Health and Care Excellence (NICE) defines Long COVID as new or ongoing symptoms 4 weeks or more after the start of acute COVID-19. Long COVID has two types of presentation, namely ongoing symptomatic COVID-19 from one to three months after onset, and post-COVID-19 syndrome for effects that persist three months or more after onset [9]. Despite the increasing number of reports, the pathophysiological mechanisms underlying Long COVID remain largely unknown, but available data indicate the multi-system nature of COVID-19 with immune dysregulation, autoimmunity, and SARS-CoV-2 neurotropism as potential reasons [4].

In the available literature, the prevalence of this syndrome is very heterogeneous, which may be the result of the diversity of the studied populations and the observation periods [2,3,4,5]. Persistent symptoms appear to have a significant impact on patients’ quality of life and return to daily activities and work. A review of 39 studies found that reduced quality of life was reported in 57% of patients with symptoms lasting more than 12 weeks [10]. Little is also known about the potential predictors of Long COVID complications, especially in healthy individuals who had recovered from COVID-19. Thus, the aim of the study was to assess the predictors of long-lasting symptoms in patients without comorbidities suffering from COVID-19.

## 2. Materials and Methods

The observational study involved 701 consecutive patients assessed in ambulatory medical care in 2020–2022, 488 of whom completed a 3-month follow-up. The patients included in the Polish Long COVID Cardiovascular (PoLoCOV-CVD) Study are part of the STOP-COVID registry (ClinicalTrials.gov identifier—NCT05018052). The final group was divided into 218 patients diagnosed with Long COVID (LC) and 270 patients without any symptoms after SARS-CoV-2 recovery (non-LC group).

LC was recognized based on the approved WHO definition [1]. Long COVID was recognized when, after a period of 3 months after COVID-19, new symptoms that appeared during the acute phase remained and/or worsened. Symptoms after COVID-19 were assessed during the patient’s interview at the follow-up visit.

The Polish Long-COVID Cardiovascular (PoLoCOV-CVD) study was a prospective, non-interventional study, which was conducted among consecutive ambulatory primary care patients in Poland. Approval from the Bioethics Committee of Lodz Regional Medical Chamber (K.B.-0115/2021) to conduct the study was obtained. Based on data from the Polish Long-COVID Cardiovascular (PoLoCOV-CVD) study, we assessed the predictors of Long COVID in healthy individuals aged 18 years and older, without comorbidities, diagnosed with COVID-19, after full recovery (resolution of clinical symptoms, minimum 14 days after last symptoms) regardless of hospitalization. All subjects included in the study were informed in detail about the research and gave their written consent to participate in it.

There were the following inclusion criteria in this study: (1) age ≥ 18 years; (2) confirmed diagnosis of COVID-19, in accordance with the current guidelines of the Ministry of Health of Poland; full recovery (resolution of acute clinical symptoms, minimum 14 days after last symptoms). Any comorbidity except obesity was considered an exclusion criterion.

Patient information, the course of the disease, post-COVID-19 complaints, and comorbidities were collected at visit “0” within 12 weeks after the end of COVID-19. A thorough clinical history and physical assessment were conducted for all patients at the outpatient clinic. Information was obtained on the presence or absence of COVID-19 symptoms, general symptoms, and the nature of symptoms if any. All patients were also ordered the following tests: (1) 12-lead electrocardiogram (ECG); (2) 24-h Holter ECG monitoring; (3) 24-h ambulatory blood pressure monitoring (ABPM), (4) echocardiography, within which quantitative measures were performed in accordance with European Society of Cardiology (ESC) guidelines—left ventricular (LV) volumes and ejection fraction (EF) were derived according to the modified biplane Simpson’s rule and right ventricular (RV) functional measures were tricuspid annular plane systolic excursion (TAPSE); and (5) biochemical tests: lipidogram (total cholesterol, LDL (low-density lipoprotein) cholesterol, HDL (high-density lipoprotein) cholesterol, triglycerides).

Participants were also grouped based on the severity at the time of initial COVID-19 diagnosis using definitions of severe COVID-19 course [1,11]:

- One of the following: hospitalization with diagnosis: pneumonia, respiratory failure, assisted breathing, or thromboembolic complications during hospitalization.
- Home course with symptoms lasting >14 days, subjective evaluation by the patient as severe (“3” on a scale of 1–3), with temperature > 38 °C, dyspnea, or saturation below 94 lasting more than 3 days.

### Statistical Analysis

We assessed the normal distribution using the Shapiro–Wilk test. The results were presented as the mean ± standard deviation or median (25–75th percentile) in the case of continuous variables, as appropriate, and as proportions for the categorical variables. Comparisons between groups were performed with the use of Student’s t-test in the case of independent variables and the Mann–Whitney U test or χ^2^ test with Yates’s correction, as appropriate. The association between demographic and clinical data and the presence or absence of persistent COVID-19 symptoms was performed using univariate and stepwise multivariable logistic regression models. The prediction of the development of Long COVID was performed with the receiver operating characteristic (ROC). The sensitivity and specificity for each point were calculated to predict the presence of persistent COVID-19 symptoms. The area under the curve was calculated and the optimal cut-off value was set as the minimum distance from the point (0,1)—the upper left corner. For all calculations, *p*-values < 0.05 were considered statistically significant. Statistical analyses were performed using the software Statistica v. 13 (TIBCO Software Inc., Palo Alto, CA, USA).

## 3. Results

### 3.1. Evaluation of Basic Characteristics

In total, 488 patients were finally included in the study (63% women and 37% men with an average age of 45.74 (SD ± 12.48)). The patients were divided into the LC group (218) with an average age of 46.03 (SD ± 11.88) and 270 patients with an average age of 44.30 (SD ± 12.82) without LC. In the LC group, there were more women (women: 69 vs. 56%, *p* = 0.003). In LC patients compared to the no LC group, significant differences regarding weight, height, and body mass index were observed. There was no significant difference in the vaccination rate against flu within the year before COVID-19 between groups (3.17% vs. 1.67%; *p* = 0.50). The clinical characteristics of the studied groups are presented in Table 1.

### 3.2. Evaluation of the Course and Symptoms during and Post COVID-19

In patients with a severe course of COVID-19 in the acute phase of the disease, Long COVID was observed more often: 34% vs. 19% (*p* = 0.0001). Moreover, when analyzing the symptoms occurring during COVID-19 disease in the LC group, more often the following symptoms were reported compared to controls: dyspnea (48% vs. 33%, *p* = 0.001), significant fatigue (72% vs. 63%, *p* = 0.034), chest pain (50% vs. 36%, *p* = 0.003), leg muscle pain (41% vs. 32%, *p* = 0.05), headache (66% vs. 52%, *p* = 0.003), arthralgia (44% vs. 25%, *p* = 0.0001), and chills (34% vs. 25%, *p* = 0.039). The number of symptoms was significantly greater in the LC group than in the control group [median 5 (range: 2–8) vs. median 2 (range: 1–5), *p* = 0.0001]. The assessment of the course and COVID-19 symptoms in the investigated groups are presented in Table 2.

The most frequently presented symptoms in the LC group were weakness (73%), impaired exercise tolerance (more than 65%), palpitations (54%), memory and concentration disturbances (more than 53%), and chest pain (44%). The assessment of symptoms that appeared 1 to 3 months post-COVID-19 in the LC group is presented in Table 3.

### 3.3. Evaluation of the Echocardiographic, Holter and Biochemical Parameters in Both Groups

No statistically significant difference was observed regarding the lipidogram, 24-h systolic and diastolic BP, and echocardiographic parameters. The LC group had a higher 24-h mean heart rate (77 [72–83] vs. 75 [70–81], *p* = 0.021). Detailed data are presented in Table 4.

### 3.4. Multivariate Regression Analysis

**Figure 1:**
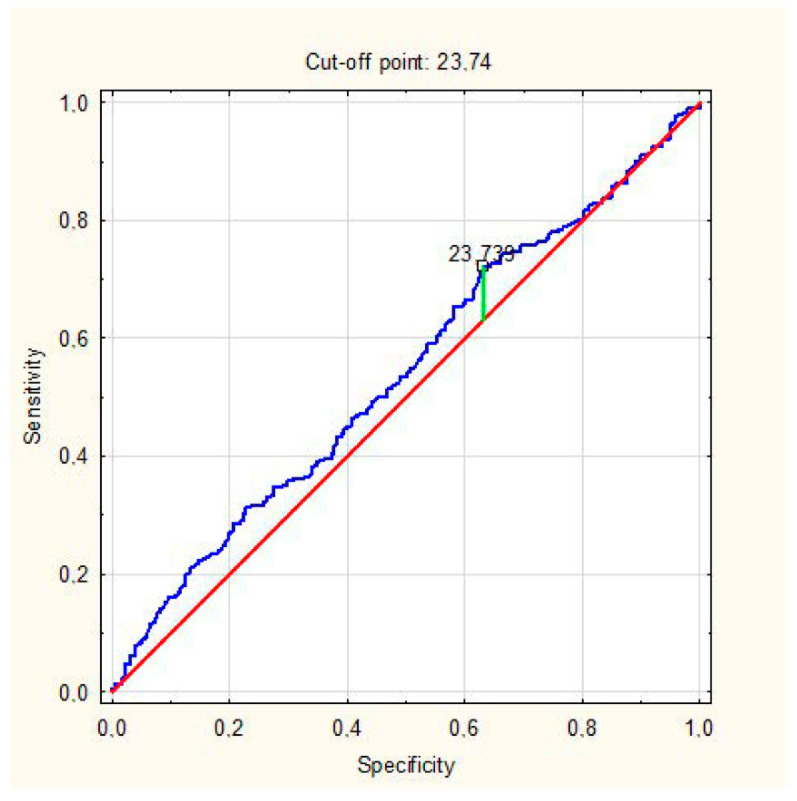
ROC analysis for the prediction of LC development by BMI. AUC = 0.541 (0.492–0.589), *p* = 0.099.

Multivariate regression analysis showed that LC patients had higher BMIs (odds ratio (OR): 1.06, 95% confidence intervals [CI] 1.016–1.100, *p* = 0.007) and almost twice as often had a severe course of COVID-19 (OR: 1.736, CI 1.071–2.814, *p* = 0.025) and presented with arthralgia in the acute phase (OR: 1.901, CI 1.225–2.950, *p* = 0.004). Based on the ROC curve, the cut-off BMI point of the increased risk of Long COVID was 23.74 kg/m^2^ (Figure 1). Logistic regression showed that a BMI higher than 23.74 kg/m^2^ predicted the development of LC (OR: 1.46, 95% CI 1.019–2.091, *p* = 0.035).

## 4. Discussion

Little is known about the lasting effects of SARS-CoV-2 infection in survivors of COVID-19. In one in five patients, regardless of the severity of the infection, symptoms after COVID-19 may last ≥5 weeks, while in one in ten patients, symptoms may persist for ≥12 weeks [12]. In our study, performed on 488 patients without any comorbidities, we noticed that patients with a severe course of the acute phase of COVID-19 developed Long COVID significantly more often. Additionally, a high BMI (≥23.74 kg/m^2^) and arthralgia during the acute phase of COVID-19 are significant risk factors for LC syndrome in healthy individuals. To the best of our knowledge, this is the first analysis that assesses the risk factors of LC in healthy subjects—those without any comorbidities pre and during COVID-19. Considering that SARS-CoV-2 infection mostly affected healthy young (in our study, the average age was only 44–46 years) individuals, these results are of special importance.

In a recent meta-analysis, the overall prevalence of COVID-19 survivors exhibiting at least one post-COVID-19 symptom was approximately 80%, with fatigue (58%) and dyspnea (24%) among the top five post-COVID-19 symptoms [13]. Huang et al. revealed that most patients in the post-COVID-19 period developed arthralgia, weakness, muscle ache, fatigue, or myalgia [14]. The study by Banić et al. revealed that out of 261 patients, after four weeks post-COVID-19, almost 80% of patients had impaired functional status. Similarly, as in our study, women were at greater risk of developing greater functional impairment in Long COVID. The malignant disease also presented a risk factor for greater functional impairment [15].

In a systematic review including 25 observational studies considering 5440 participants, the frequency of Long COVID ranged from 4.7% to 80%, and the criteria used to define Long COVID did not meet the WHO definition. The most prevalent symptoms were chest pain (up to 89%), fatigue (up to 65%), dyspnea (up to 61%), and cough and sputum production (up to 59%). Potentially associated risk factors of Long COVID in this study were female sex, old age, severe clinical status, a high number of comorbidities, hospital admission, and oxygen supplementation in the acute phase [16]. In the Chinese study, the long-term health consequences after COVID-19 were assessed approximately 186 days after symptom onset in 1733 patients (52% men). Fatigue or muscle weakness (63%) and sleep difficulties (26%) were the most common symptoms. Additionally, 23% of patients reported anxiety or depression at follow-up visits. Patients who were more severely ill during their hospital stay had more severe impaired pulmonary diffusion capacities and abnormal chest imaging manifestations. In a multivariable analysis, women and participants with a severity scale of 5–6 had a higher risk of lung diffusion impairment, anxiety or depression, and fatigue or muscle weakness [17]. Moreover, in our study, female sex and a severe course of COVID 19 were predictors of long-lasting post-COVID-19 symptoms.

In the retrospective study by Osikomaiya et al. [18], in 274 patients attending the COVID-19 outpatient clinic in Lagos State, more than one-third (41%) had persistent COVID-19 symptoms after discharge, and approximately 20% had more than three persistent COVID-like symptoms. The most persistent COVID-like symptoms experienced were easy headaches (13%), fatigability (13%), and chest pain (10%). There was no association between sex, age, and a history of hypertension, diabetes, or multiple comorbidities with the presence of persistent COVID-19-like symptoms. Further, there was similarly to our study regarding a significant association between the severity of COVID-19 disease at initial diagnosis and Long COVID occurrence after hospitalization [18]. The most common symptoms manifested by survivors in our study’s Long COVID group were weakness (73%), impaired exercise tolerance (66%), dyspnea (32%), anosmia and ageusia (24%), excessive sweating (29%), chest pain (44%), muscle pain (25%), ascites (swelling) (12%), varicose veins of lower extremities (6%), skin lesions (11%), hair loss (28%), palpitations (54%), syncope (4%), memory and concentration disturbances (54%), headache (34%), and arthralgia (2%). Our findings align with reports on COVID-19 symptoms in survivors, especially regarding weakness based on recent studies [16,17].

Previously, 438 COVID-19 survivors from Wuhan, China, after three months or more from hospital discharge, were assessed regarding post-COVID symptoms [19]. The persistent symptoms were common, including general symptoms (50%), respiratory symptoms (39%), cardiovascular-related symptoms (13%), psychosocial symptoms (22.7%), and alopecia (28.6%). The authors found that symptoms such as physical decline/fatigue (*p* < 0.01), post-activity polypnea (*p* = 0.04), and alopecia (*p* < 0.01) were more common in female compared to male subjects. Dyspnea during hospitalization was associated with subsequent physical decline or fatigue, post-activity polypnea, and a resting heart rate increase. The survivors with a physical decline or fatigue or post-activity polypnea had a longer duration of virus shedding after COVID-19 and a longer length of hospital stay than those without these symptoms. Our LC group had a significantly higher mean heart rate in 24 h ECG ambulatory monitoring after the 3-month follow-up. The study of Qiutang Xiong et al. [19] found that 13% of patients had cardiovascular symptoms, the most important of which was increased resting heart rate. A history of pulse ≥ 90 bpm during hospitalization was associated with a resting heart rate increase in convalescence. In addition, seven patients reported a recent diagnosis of hypertension after a COVID-19 infection. Likewise, in a study by Lewek et al. [20], the post-COVID-19 complications appeared 4 to 16 weeks after disease recovery. Severe cardiovascular complications were observed in approximately 27% of hospitalized patients. The patients with severe complications had a significantly higher decrease in the ejection fraction (36% vs. 0%, *p* < 0.001), prevalence of diabetes (36 vs. 8%; *p* = 0.01), higher resting heart rate at admission (85 vs. 72 bpm; *p* < 0.001), and higher levels of troponin T (17.9 vs. 4.2 pg/mL; *p* = 0.01), C-reactive protein (*p* = 0.02), and troponin T (17.9 vs. 4.2 pg/mL; *p* = 0.01). These findings may be evidence of COVID-19’s long-term damage to the cardiovascular system.

The relationship between COVID-19 severity and Long COVID may be explained by the immune response to the SARS-CoV-2 virus, which stimulates the production of cytokines and other inflammatory mediators [21,22,23,24]. The multi-systemic inflammatory response to the virus may also be responsible for persistent COVID-19 symptoms in survivors [24,25,26,27]. Other likely mechanisms of Long COVID include vascular inflammation, detected using 2-deoxy-2-[18F] Fluoro-D-glucose [26], or endothelial dysfunction caused by the action of the SARS-CoV-2 on ACE2 receptors [26,27,28,29].

Gavriilaki et al. [30] and Bonaventura et al. [31], in their studies, emphasized that endothelial dysfunction may play a key role in infection with the SARS-CoV-2 virus. The impaired function of the vascular endothelium is associated with the expression in the vascular system of the proinflammatory, pro-atherosclerotic, and pro-thrombotic phenotype, which is directly related to the development of cardiovascular diseases [30]. In addition, an association between endothelial dysfunction and abnormal vasodilation was observed, which may cause changes in hemodynamic parameters, such as an increase in heart rate in patients, which was also confirmed by the results of our research. Even though, in our work, we did not measure the heart rate value before patients became ill with COVID-19, higher values of the tested parameter were observed in the Long COVID group.

Several studies [32] have reported that obesity is associated with adverse outcomes after SARS-CoV-2infection, but most studies included patients admitted to hospital with COVID-19 symptoms. There are some data that indicate that not only obesity but also excess weight (i.e., a BMI of >25 kg/m^2^) is related to a worse course of COVID-19. In the study of Gao et al., the hazard ratio of severe outcomes from COVID-19 (i.e., admission to hospital, admission to ICU, or death) increased progressively above a BMI of 23 kg/m^2^, which was not attributable to excess risks of related diseases such as type 2 diabetes. The relative risk due to increasing BMI was particularly notable in patients younger than 40 years and of Black ethnicity [33]. In our study, BMI (≥23.74 kg/m^2^) during the acute phase of COVID-19 increased the risk of the development of LC syndrome by 46% in healthy individuals.

In the study by Bhaskar et al. [34], the authors showed that BMI is an independent predictor of cause-specific COVID-19 mortality, and not of the caseload per million population. Countries with obesity rates of 20–30% had a significantly higher number of deaths per million population than both those in <20% and >30% slabs. In the study of Vimercati et al., in a group of 352 healthcare workers after SARS-CoV-2 infection, including 168 with Long COVID, the LC group showed mean BMI values higher than in the non-LC group (25.9 kg/m^2^ vs. 24.8 kg/m^2^, *p* = 0.02). Moreover, similarly to our study, participants with overweight (OR = 1.6 CL 95%: 1.05–2.56; *p*-value = 0.029) had an increased risk of developing Long COVID [35]. The risk of severe COVID-19 outcomes attributable to excess weight has been proposed to be a consequence of metabolic impairment of organ functioning, leading to insulin resistance, as well as due to a pro-inflammatory state [36].

Another significant risk factor of Long COVID in our study was the presence of arthralgia during COVID-19. Viral infections are a known cause of acute arthralgia and arthritis; monoarticular arthritis can occur after infection by various pathogens [37]. COVID-19 has also been found to cause reactive arthritis and new-onset inflammatory arthritis, typically occurring within four weeks after its diagnosis [38]. Peterson et al. highlighted in their study that arthralgia was the most common Long COVID symptom [39]. The example of a middle-aged woman who developed seropositive rheumatoid arthritis (RA) with an elevated CRP and erythrocyte sedimentation rate (ESR) 2 weeks post-hospital discharge after a COVID-19 infection, with previously negative tests for rheumatoid factor (RF) and anticyclic-citrullinated protein (anti-CCP), suggested COVID-19 infection as a trigger for autoimmunity [40]. The occurrence of Long COVID in patients with arthralgia during COVID-19 might arise from inflammatory and/or immune responses. This hypothesis is based on the involvement of proinflammatory markers (IL-6 and TNF-α) released in alveolar and musculoskeletal inflammation [41,42,43].

Strengths and limitations of the study. Our study was limited to interventions that were conducted in primary care. The limiting factor concerns the relatively small follow-up period of 3 months. The studies did not assess biochemical parameters, except of lipidogram. We assessed self-reported persistent COVID-19 symptoms. Patients in our study did not undergo respiratory function examinations, and the specific degree of functional decline is not clear. Instead, they only compared their perception with their previous respiratory function from a subjective perspective. However, the diagnosis was carefully made to avoid potential confounders such as symptoms that had been present before SARS-CoV-2 infection, concurrent infection from other viral agents, and underlying comorbidities.

In our study, the percentage of participants with obesity was relatively small, and there were no patients with BMI > 35 but only with obesity class I. We decided not to exclude these patients because the results presenting the association of BMI as small as 24 with Long COVID are interesting and valuable. We showed that the risk of cardiovascular complications increases not only for obese patients, not even only for those overweight, but still for those with an upper limit of the normal range of BMI (approximately 24 kg/m^2^), which makes these results novel and important from the clinical point of view. It should also be emphasized that the AUC was only slightly higher than 0.5, which is the limitation of that observation. Due to the fact that our ROC analysis was not able to clearly identify a BMI cut-off to effectively discriminate between Long COVID and non-Long COVID patients, we conducted an additional logistic regression analysis, which confirmed that patients with BMI > 23.74 kg/m^2^ were at 46% higher risk of Long COVID occurrence.

Our results are additionally of special importance now, as even 60% of patients during the pandemic have overweight and obesity, and these results should be a call for action to fight this, in order to reduce the risk of severe complications of SARS-CoV-2 infections during Long COVID.

More studies are needed to assess the predictors of persistent COVID symptoms, as well as the quality of life. It is important to distinguish between symptoms from persistent chronic inflammation and symptoms that may arise as consequences of organ damage (pulmonary fibrosis or pleural effusion) and other persistent symptoms from factors such as a long hospital stay and social isolation (nutritional anemia, weight loss, and muscle wasting) [44]. So far, the validity of screening for cardiovascular complications in patients after COVID-19 has not been confirmed. Until appropriate recommendations are made, the diagnostic procedure should be carried out in an individualized manner, based on the course of the acute phase of COVID-19 and clinical symptoms reported or presented after COVID-19 based on the clinical phenotype. Based on our results in patients after COVID-19 without comorbidities, an ambulatory assessment might be indicated mainly in women with obesity and/or arthralgia, after a severe course of infection. There is an urgent need to understand this emerging, complex, and challenging medical condition [45].

## 5. Conclusions

In conclusion, to the best of our knowledge, our study is the first to report the possible risk factors of Long COVID in COVID-19 survivors without any comorbidities. A severe course of COVID-19, BMI, and arthralgia are independently associated with the risk of Long COVID in healthy individuals.

## Figures and Tables

**Table 1:** The clinical characteristics—differences between groups with and without LC.

	Long COVID (*n* = 218)	No Long COVID (*n* = 270)	*p*	Total (*n* = 488)
Clinical Characteristics				
Age	46.03 ± 11.88	44.30 ± 12.82	0.13	45.74 ± 12.48
Women	69.14%	55.96%	**0.003**	308 (63.11%)
Men	30.86%	44.04%	**0.003**	179 (36.68%)
Weight [kg]	82 (68–95)	72 (63–85)	**0.000**	78 (65–90)
Height [cm]	171 (164–176)	167 (161–173)	**0.001**	168 (163–176)
Body Mass Index [kg/m²]	28 (24–31)	26 (23–30)	**0.0001**	26 (24–31)
Vaccine against flu last year	3.17%	1.67%	0.50	11 (2.55%)

**Table 2:** The assessment of the course and COVID-19 symptoms in both groups.

	Long COVID (*n* = 218)	No Long COVID (*n* = 270)	*p*	Total (*n* = 488)
**The course of COVID-19**				
Symptomatic course of COVID-19 (yes)	98.82%	96.34%	0.15	435 (97.75%)
Home isolation	91.39%	92.45%	0.67	440 (91.86%)
Hospitalization without pneumonia	1.57%	2.15%	0.93	4 (2.15%)
Hospitalization with pneumonia	5.81%	6.42%	0.79	27 (6.07%)
Hospitalization with ICU	0.78%	0.54%	0.78	3 (0.68%)
Pneumonia COVID-19	3.91%	2.70%	0.79	15 (3.40%)
Severe course (hospital or home isolation)	34.22%	18.62%	**0.0001**	125 (27.72%)
**Symptoms during COVID-19**				
Temperature < 36.6 C deg.	12.20%	16.58%	0.19	62 (14.06%)
Temperature ≥ 36.6 and < 37.5 C deg.	26.56%	27.46%	0.83	121 (26.95%)
Temperature ≥ 37.5 C deg.	51.15%	53.61%	0.60	238 (52.19%)
Cough	60.08%	54.26%	0.22	257 (57.62%)
Dyspnoea	48.26%	32.62%	**0.001**	186 (41.70%)
Upper respiratory tract infection/rhinitis	27.06%	30.48%	0.43	126 (28.51%)
Influenza-like symptoms	47.06%	41.18%	0.22	197 (44.57%)
Anosmia	9.88%	12.37%	0.41	48 (10.93%)
Ageusia	5.53%	5.98%	0.84	25 (5.72%)
Anosmia or ageusia	52.51%	48.45%	0.39	230 (50.77%)
Significant fatigue	72.27%	62.89%	**0.034**	307 (68.22%)
Chest pain	50.20%	35.87%	**0.003**	194 (44.19%)
Back muscle pain	54.15%	47.40%	0.16	228 (51.24%)
Leg muscle pain	40.87%	31.72%	**0.05**	162 (36.99%)
Headache	66.27%	52.36%	**0.003**	269 (60.31%)
Arthralgia	43.65%	24.46%	**0.0001**	155 (35.55%)
Diarrhea	21.74%	18.92%	0.47	90 (20.55%)
Vomiting	7.54%	5.43%	0.38	29 (6.65%)
Chills	33.60%	24.46%	**0.039**	130 (29.75%)
BP elevation or dysregulation of previously well-controlled BP	8.73%	6.49%	0.39	34 (7.78%)
Impaired hearing	5.95%	4.89%	0.63	24 (5.50%)
Sum of symptoms	5 (2–8)	2 (1–5)	**0.0001**	4 (2–6)

Abbreviations: BP—blood pressure.

**Table 3:** The assessment of symptoms in LC group.

	Long COVID (*n* = 218)
Weakness	72.80%
Impaired exercise tolerance	65.88%
Palpitations	54.15%
Memory and concentration disturbances	53.75%
Chest pain	44.09%
Headache	34.39%
Dyspnoea	32.02%
Excessive sweating	29.25%
Hair loss	28.06%
Muscle pain	24.60%
Anosmia and ageusia	24.12%
Cough	23.23%
Raynaud syndrome	14.29%
Ascites (swelling)	11.51%
Skin lesions	10.67%
Conjunctivitis	8.30%
Varicose veins of lower extremities	6.35%
Neurological disturbances	5.88%
Syncope	3.57%
Arthralgia	1.55%
Stenocardia	0%

**Table 4:** Differences between groups with and without Long COVID among patients without comorbidities.

	Long COVID (*n* = 218)	No Long COVID (*n* = 270)	*p*	Range
**Echocardiography**				
LA (mm)	37 (35–40)	37 (34–40)	0.47	37 (34–40)
AD (mm)	30 (28–32)	30 (27–32)	0.99	30 (28–32)
RV (mm)	28 (26–30)	28 (26–30)	0.17	28 (26–30)
EF (%)	60 (58–60)	60 (60–60)	0.13	60 (60–60)
TAPSE (mm)	25 (24–25)	25 (24–26)	0.24	25 (24–25)
LVMass (g/m²)	169 (127–204)	166 (136–212)	0.47	167 (132–210)
LVESd (mm)	30 (26–35)	31 (28–34)	0.19	30.5 (27–34)
LVEDd (mm)	45 (42–48)	45 (42–48)	0.42	45 (42–48)
IVSs (mm)	13 (12–14)	13 (12–14)	0.44	13 (12–14)
IVSd (mm)	9 (9–11)	10 (8–11)	0.42	10 (9–11)
**24 h ECG ambulatory monitoring**				
Mean HR	77 (72–83)	75 (70–81)	**0.021**	76 (71–82)
Max HR	180 (153–206)	180 (155–214)	0.45	180 (155–211)
Min HR	47 (42–53)	46 (42–50)	0.39	48 (42–52)
**ABPM**				
MAP mean daily	92 (86–97)	92 (86–98)	0.06	92 (86–97)
MAP mean day	96 (90–103)	95 (89–103)	0.80	96 (89–103)
MAP mean night	80 (74–86)	79 (74–84)	0.49	30 (20–42)
Systolic dipping	14.09 ± 6.98	14.87 ± 6.81	0.30	14.42 ± 7.04
Diastolic dipping	17.18 ± 8.27	17.12 ± 7.48	0.94	17.19 ± 7.80
**Biochemical parameters**				
TC	193 (166–216)	198 (169–221)	0.32	194 (169–220)
HDL	57 (48–64)	59 (49–64)	0.85	57 (47–62)
LDL	114.80 ± 34.55	123.32 ± 38.88	0.06	118 (95–139)
TG	97 (66–143)	88 (61–134)	0.15	95 (66–143)

Abbreviations: LA—left atrial; AD—aortic diameter; RV—right ventricle; EF—ejection fraction; TAPSE—tricuspid annular plane systolic excursion; LVMass—left ventricular mass; LVESd—left ventricular end-systolic diameter; LVEDd—left ventricular end-diastolic diameter; IVSs—interventricular septal end systolic thickness; IVSd—interventricular septal end diastolic thickness; HR—heart rate; MAP—mean arterial pressure; TC—total cholesterol; HDL—high-density lipoprotein; LDL—low-density lipoprotein; TG—triglycerides.

## Data Availability

The data underlying this article cannot be shared publicly for the privacy of individuals that participated in the study.
